# Macro Responsibility in the Microvascular World: Nurse Experiences in Flap Care, a Phenomenological Study

**DOI:** 10.3390/healthcare14121808

**Published:** 2026-06-22

**Authors:** Dilay Hacıdursunoğlu Erbaş, Evin Korkmaz

**Affiliations:** 1 Nursing Department, Faculty of Health Sciences, Sakarya University of Applied Sciences, 54050 Sakarya, Türkiye; 2 Independent Researcher, 35430 İzmir, Türkiye

**Keywords:** free tissue flap, flap monitoring, care, microsurgery, postoperative care, qualitative research

## Abstract

**Highlights:**

**What are the main findings?**

- Experiences of nurses involved in microvascular free flap monitoring and postoperative flap care were explored through semi-structured interviews. Participants emphasized the intensive monitoring requirements of flap care, the importance of clinical observation and experience, and the emotional burden associated with responsibility for flap viability.
- Four main themes were identified, highlighting: (1) clinical monitoring and evaluation in the care process, (2) challenges and difficulties encountered during postoperative monitoring, including workload and lack of standardized protocols, (3) emotional and professional reflections related to responsibility and stress, and (4) suggestions for improving flap care through education, standardized tools, and multidisciplinary collaboration.

**What are the implications of the main findings?**

- Despite advances in microsurgical reconstruction, flap monitoring practices continue to rely heavily on individual clinical experience and observational skills, indicating the need for more standardized and structured monitoring approaches.
- Findings highlight the importance of evidence-based monitoring protocols, multidisciplinary teamwork, and targeted education programs to strengthen nurses’ competencies, support early detection of vascular compromise, and improve the quality and safety of postoperative flap care.

**Abstract:**

**Background/Objectives**: Postoperative monitoring of microvascular free flaps is critical for early detection of vascular complications and flap survival. Nurses play a central role in this process; however, qualitative evidence on their experiences and challenges remains limited. This study explored nurses’ experiences in free tissue flap care to identify clinical practices, challenges, and improvement needs. **Methods**: A phenomenological qualitative design was used. Data were collected through semi-structured interviews with nine nurses experienced in free tissue flap care, recruited via purposive and snowball sampling. Interviews were conducted online and lasted 30–45 min. Data were analyzed using content analysis with MAXQDA 2025. Inter-researcher reliability was 97%. **Results**: The findings were categorized into four main themes and seventeen subthemes: (1) clinical monitoring and evaluation in the care process, (2) challenges and difficulties, (3) emotional and professional reflections, and (4) suggestions for improving care. Nurses reported that flap care requires intensive monitoring, rapid decision-making, and close collaboration with physicians, especially within the first 24–48 h. Monitoring was largely based on observation and experience due to the lack of standardized protocols. Major challenges included high workload, frequent assessments, and donor site management. Emotional burden, stress, and responsibility were also prominent. **Conclusions**: Free flap care is a complex and demanding process for nurses. The lack of standardized monitoring tools and protocols is a key gap. Developing structured tools, improving training, and strengthening multidisciplinary collaboration may enhance patient safety and care quality.

## 1. Introduction

Microvascular free flaps are well-established and reliable surgical techniques widely used in reconstructive procedures, including head and neck, breast, and extremity reconstruction [1,2]. With advancements in surgical techniques and microsurgical equipment, success rates in free flap surgery have exceeded 95% [3]. Despite these advancements, vascular complications arising in the early postoperative period remain among the leading causes of flap loss [4].

A significant proportion of free flap failures results from circulatory disorders caused by factors such as arterial or venous thrombosis, vessel kinking, hematoma formation, or external compression. These complications most commonly occur in the early postoperative period, with the first 48–72 h representing the period of highest risk for flap viability [3]. Therefore, effective early flap monitoring significantly increases flap salvage rates by enabling rapid detection of vascular insufficiency and timely surgical intervention [2]. This demonstrates the critical role of postoperative flap monitoring in the success of microsurgical reconstruction. Early diagnosis and prompt intervention in flap monitoring are considered key factors determining flap survival.

Currently, clinical evaluation remains the most widely used method for postoperative flap monitoring. This approach involves assessing parameters such as flap color, temperature, capillary refill time, tissue turgor, and Doppler signals. Clinical monitoring is regarded as the “gold standard” in many centers due to its simplicity, rapid applicability, and cost-effectiveness [2,5,6,7]. However, clinical assessment is largely based on observation and experience and is open to subjective interpretation. This can lead to variations in evaluation among different clinicians. In recent years, significant studies have been conducted to develop objective methods for flap monitoring. Many auxiliary monitoring methods, such as implanted Doppler probes, near-infrared spectroscopy, laser Doppler flowmetry, microdialysis, and various imaging techniques, have been described in the literature. Despite their potential advantages, most of these methods have not become widespread in routine clinical practice due to factors such as high cost, technical complexity, and limited accessibility [2,6,8].

Flap care involves close postoperative monitoring of flap viability through serial clinical assessments of flap color, temperature, capillary refill, tissue turgor, bleeding characteristics, and Doppler signals [5,9,10]. Currently, the combined use of clinical examination and handheld Doppler sonography is widely accepted as a standard approach in postoperative free flap monitoring because it is rapid, non-invasive, low-cost, and practical [9]. However, the clinical assessment process requires experience and advanced observational skills; therefore, variations in flap appearance, warning signs, assessor expertise, and institution-specific protocols may lead to differences in clinical evaluation [5,9]. Furthermore, perioperative care and monitoring protocols for patients undergoing free flap procedures vary considerably across institutions, and there is generally no consensus on this issue [10,11]. In this context, nurses play a critically important role in free flap monitoring. In the postoperative period, most flap monitoring is performed by nurses, and their practices, knowledge, and clinical judgment play a crucial role in the early detection and prevention of potential complications [9]. The literature indicates that nurses’ knowledge and practices regarding flap monitoring may vary between institutions, and there is a lack of standardization in monitoring protocols [10].

A significant portion of current flap monitoring approaches relies on clinical observations, and evaluations are largely based on subjective interpretation. This reduces the objectivity of flap assessment and can make it more difficult to recognize early complications. The aim of this study was to explore the lived experiences of nurses involved in the care of patients who underwent free tissue flap procedures, with a particular focus on clinical monitoring practices, decision-making processes, challenges, emotional and professional reflections, and suggestions for improving care. Additionally, this study aimed to make nursing practices related to flap care more visible and thereby contribute to improving the quality and safety of patient care.

Research questions: What are the experiences of nurses caring for patients who have undergone free tissue flap procedures? Under what themes are the experiences of nurses caring for patients who have undergone free tissue flap procedures categorized?

## 2. Materials and Methods

### 2.1. Research Design

This study was conducted using a descriptive phenomenological qualitative research design. The descriptive phenomenological approach was selected because the study aimed to explore and describe nurses’ lived experiences of postoperative free tissue flap care as directly as possible [12]. This approach allowed the researchers to focus on participants’ descriptions of their clinical experiences, perceptions, challenges, and care-related meanings within the context of flap monitoring. The Consolidated Criteria for Reporting Qualitative Research (COREQ) were used as a guideline for reporting the study [13].

### 2.2. Sample and Participants

The population of the study consisted of nurses across Türkiye who had experience in providing care for patients undergoing free tissue flap procedures. Participants were selected using purposive sampling and snowball sampling. Purposive sampling was used to recruit nurses who could provide rich and relevant information about postoperative free flap care because of their direct clinical experience in this field. Snowball sampling was also used because nurses with free flap care experience represent a relatively specific professional group, and referrals from eligible participants facilitated access to other nurses with similar clinical experience. The sampling criteria were as follows: providing free tissue flap care for at least one year, actively working in this field, being open to communication, and agreeing to participate in the study. Nurses who had no direct experience in postoperative free flap monitoring, who were not actively involved in patient care related to flap procedures, or who did not provide informed consent were excluded from the study. The research was completed with nine nurses, at which point data saturation was reached.

### 2.3. Data Collection Tools and Process

Data were collected between June and July 2025 using a semi-structured interview form consisting of open-ended questions. The interview guide was developed by the researchers based on the aim of the study, the descriptive phenomenological approach, relevant literature on postoperative free flap monitoring and nursing care, and the researchers’ clinical and academic expertise in surgical nursing. The questions were designed to elicit nurses’ lived experiences of flap care, including their clinical monitoring practices, decision-making processes, perceived challenges, emotional and professional reflections, and suggestions for improving care.

The interviews were conducted individually via video conferencing on online platforms, considering physical distance and time constraints. Each interview lasted approximately 30–45 min. Data saturation was assessed during the data collection and preliminary analysis process. After each interview, the researchers reviewed the data and compared emerging codes and themes with those obtained from previous interviews. Data collection was continued until no new codes, categories, or themes emerged from the interviews. Saturation was considered to have been reached after the ninth interview, when the final interviews repeated previously identified concepts and did not provide substantially new information.

The semi-structured interview form used for data collection in the study comprised the following questions:

1. How does the care process begin for patients for whom you perform free-tissue flap monitoring? What are your initial assessment steps?
2. Could you describe in detail the steps you follow during the flap care process?
3. Which clinical indicators do you evaluate during follow-up? (e.g., color, temperature, capillary refill, pulse, etc.)
4. Do you use any tools or special techniques during flap monitoring?
5. What is your initial response when encountering suspicious findings? How do you communicate with physicians in such situations?
6. What are the most challenging aspects of this process for you?
7. When making decisions during the follow-up process, which factor do you prioritize: knowledge, experience, or protocols?
8. How does the flap care process affect you emotionally and professionally?
9. In your opinion, what are the most important factors affecting patient safety and quality of care during this process?
10. If a follow-up form or checklist were to be developed, what should it include?
11. Which aspects of nursing practice related to flap care do you think require improvement?

### 2.4. Data Analysis

The interview data were analyzed using an inductive content analysis approach. All interviews were transcribed verbatim and read several times by the researchers to gain familiarity with the data. Meaningful statements related to nurses’ experiences of free tissue flap care were identified and coded. Similar codes were grouped into categories, and these categories were then organized into subthemes and main themes. MAXQDA 2025 was used to support systematic coding, data organization, and theme development. Throughout the analysis process, the researchers repeatedly returned to the transcripts to ensure that the codes, categories, subthemes, and themes were grounded in participants’ statements. Direct quotations were used to support the findings and reflect participants’ experiences in their own words.

### 2.5. Ethical Approach and Data Security

The study was conducted in accordance with the Declaration of Helsinki, and approved by the Ethics Committee of Sakarya University of Applied Sciences, with ethics approval number 57/17 dated 30 May 2025. Informed consent was obtained from all nurses involved in the study. To ensure data security, participants were informed that audio and video recordings would be stored in password-protected (encrypted) files accessible only to the researchers and used solely for the purpose of this study. Participant confidentiality was maintained by assigning code names (e.g., N1, N2) during the analysis and reporting of the findings.

### 2.6. Researcher Reflexivity

Researcher reflexivity was considered throughout the study process. The researchers had academic and clinical experience in surgical nursing and qualitative research, which informed their sensitivity to the clinical context of postoperative free tissue flap care. To reduce the influence of prior assumptions on data interpretation, the researchers discussed their pre-understandings before and during the analysis process. During coding and theme development, the researchers repeatedly returned to the interview transcripts and participant quotations to ensure that interpretations remained grounded in the participants’ accounts. Differences in interpretation were discussed through consensus meetings, and reflexive notes were used to support transparency in analytic decisions.

### 2.7. Trustworthiness and Rigor

Trustworthiness was ensured using the criteria of credibility, transferability, dependability, and confirmability [14]. To support the rigorous conduct of the study, the researchers received training in qualitative research prior to the study. To enhance credibility, participant confirmation was obtained by playing back the interview recordings to the participants after each interview and obtaining their approval. Direct quotations were included in the findings without altering the nurses’ original statements.

To support dependability, two researchers independently coded the data. The coding framework was developed inductively from the interview data rather than being predetermined. Before independent coding, the researchers reviewed the interview transcripts and discussed the analytic approach to establish a shared understanding of the coding process. After independent coding, the resulting codes and categories were compared. Differences in coding and interpretation were discussed in consensus meetings, and the codes, categories, subthemes, and themes were finalized through agreement between the researchers. The level of agreement between the researchers was calculated using the formula [(consensus)/(consensus + disagreement)] × 100 [15,16], and the inter-researcher agreement was found to be 97%.

To enhance transferability, the context of the study, participant characteristics, sampling strategy, and data collection process were described in detail. To support confirmability, coding decisions, category development, and consensus discussions were documented throughout the analysis process.

## 3. Results

Nine nurses participated in this qualitative study. Of the participants, 88.9% were women, and their average age was 30.7 ± 6.0 years. The average professional experience was 7.9 ± 5.9 years. When asked about the clinical setting in which they provided free tissue flap care, 44.4% reported working in the plastic surgery department, and 55.6% reported receiving flap care training from physicians in their clinic (Table 1).

**Figure 1:**
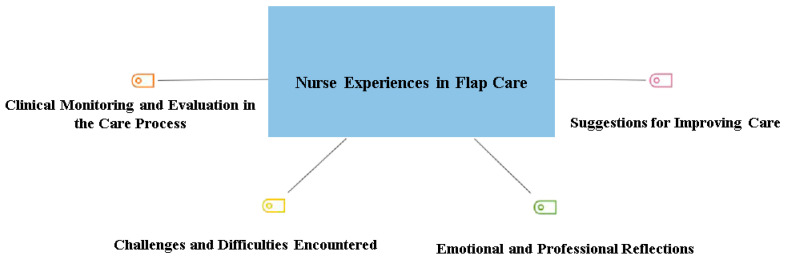
Display of themes. It was created by the authors during the analysis phase.

The participants’ views indicate that flap care is conducted through intensive clinical monitoring, relying on multidimensional and primarily observational parameters. During the interviews, nurses emphasized that flap care differs significantly from routine ward care due to its need for heightened attention, frequent assessment, and timely intervention, and that it increases workload in various ways. Participants reported experiencing both a high level of responsibility and a considerable emotional burden during the care process. Their perspectives not only describe current care practices but also include concrete and systematic suggestions for improving the flap care process. In this context, the findings of nurses’ experiences, consisting of four main themes and 17 subthemes, are presented below (Figure 1, Table 2).

### 3.1. Theme 1: Clinical Monitoring and Evaluation in the Care Process

**Figure 2:**
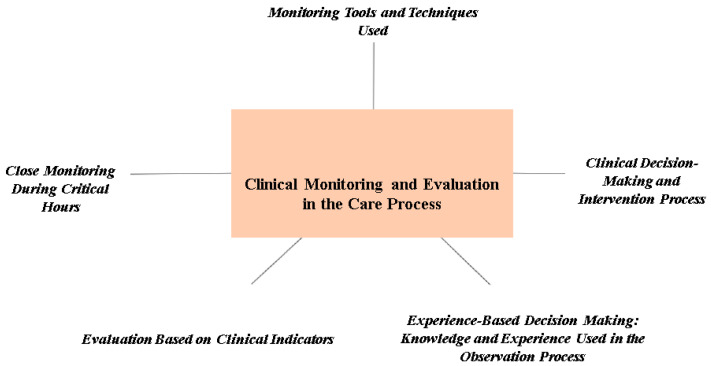
Display of theme 1. It was created by the authors during the analysis phase.

The participants’ opinions indicate that clinical follow-up in flap care is intensive, multidimensional, and primarily based on observational parameters. It was emphasized that the first 24–48 h are considered the “critical period,” during which close, frequent, and multi-parameter monitoring is performed. The follow-up process was primarily based on indicators of circulation, signs of tissue viability, and close communication with the physician. In the decision-making process, clinical experience, physician guidance, and observational findings took precedence over standardized protocols. The subthemes identified in this theme are shown in Figure 2.

#### 3.1.1. Close Monitoring During Critical Hours

This sub-theme consists of codes related to the initiation of flap care, hourly follow-up practices, immobilization, postponement of oral intake, and close monitoring of vital signs. Participants defined the first hours following flap surgery as a high-risk period and emphasized that hourly evaluation, movement restriction, and frequent monitoring of vital signs are fundamental practices during this phase. Patients were generally kept immobilized, and in some cases, mobilization and oral intake were delayed in accordance with physician orders. The care approach during this period was described as “similar to intensive care.”

*“After returning from surgery, we perform hourly flap monitoring for the first 24 h; it’s very important.”*

(N5)

*“Since the first 24–48 h are critical, we monitor the flap hourly.”*

(N3)

*“We do not mobilize the patient on the first day; we keep them completely immobile.”*

(N7)

*“Oral intake is generally not started for 24 h in case circulation is compromised and the patient needs to be taken back to surgery.”*

(N6)

#### 3.1.2. Evaluation Based on Clinical Indicators

This sub-theme includes codes related to color assessment, temperature measurement, capillary refill, Doppler pulse monitoring, pulse palpation, hematoma/bleeding control, edema monitoring, coolness and paleness, and tissue viability. Participants stated that flap monitoring is primarily based on indicators of circulation and perfusion. The most frequently emphasized parameters are color, temperature, and Doppler pulse monitoring. It was observed that assessment is generally based on a combination of observation, palpation, and Doppler auscultation. Some participants also reported using a non-contact thermometer to measure local temperature.

*“Color and temperature are very helpful for us, and we also check the pulse with Doppler.”*

(N1)

*“If there is coldness or paleness, we consider that circulation might be impaired.”*

(N9)

*“We slightly prick with a needle tip to see if there is any bleeding and check for circulation.”*

(N8)

*“If it drops below 36 degrees, we consider it a risk.”*

(N4)

#### 3.1.3. Experience-Based Decision Making: Knowledge and Experience Used in the Observation Process

This subtheme consists of codes related to learning through observation, learning from physicians, experience, the integration of education and experience, and learning through personal research. Participants stated that the decision-making process in flap care is largely based on experience and that, in the absence of standardized nursing protocols, clinical observation and experience play a guiding role. It was observed that most learning occurs during clinical practice and follows a “master-apprentice model.”

*“There is no protocol we follow, we got used to it as we saw the cases.”*

(N1)

*“I haven’t received any special training, I learned through my own research.”*

(N7)

*“We learn entirely through experience.”*

(N8)

*“It all depends on experience and practice.”*

(N3)

#### 3.1.4. Clinical Decision-Making and Intervention Process

This sub-theme includes codes related to preliminary assessment, the nurse-head nurse-physician communication chain, notification through photographs, video consultation, procedures followed in cases of suspicion, and rapid intervention. Participants described a hierarchical and rapid communication process in response to suspicious findings. The initial assessment was conducted by the nurse, followed by notification of the head nurse and physician. The use of photograph sharing and real-time visual communication was also noted. It was emphasized that interventions are typically carried out promptly.

*“In case of an abnormal situation, we immediately inform the physician.”*

(N5)

*“We take a photo and send it; the physician evaluates it.”*

(N8)

*“Even hours later, if the circulation worsens, they can be taken in for surgery again.”*

(N6)

*“If I can’t get a pulse, we immediately inform them.”*

(N3)

*“Any changes are addressed immediately.”*

(N7)

#### 3.1.5. Monitoring Tools and Techniques Used

This sub-theme encompasses codes related to handheld Doppler devices, including mini-Doppler devices, thermometers, photographic monitoring, visual comparison, and laboratory monitoring. Participants emphasized that the Doppler device is the most fundamental monitoring tool. In addition, non-contact thermometers, serial photography, and laboratory parameters were also included in the monitoring process. Photographic comparison was described as a systematic monitoring practice, particularly in certain clinics.

*“The most important thing is to hear the heartbeat with the Doppler.”*

(N5)

*“We have a mini-Doppler; the assistants do the monitoring, and I observe as well.”*

(N7)

*“We also monitor blood values.”*

(N6)

*“Usually, we take photos and compare them.”*

(N4)

### 3.2. Theme 2: Challenges and Difficulties Encountered

This theme encompasses the structural, organizational, and clinical challenges encountered by participants during flap monitoring and care. Nurses emphasized that flap care differs significantly from routine ward care, as it requires heightened attention, frequent assessments, and timely interventions, thereby increasing workload in various ways. In particular, the obligation for hourly monitoring, high ward occupancy, large patient volumes, and the need to remain constantly alert make the care process challenging both physically and cognitively. Additionally, factors such as prolonged fasting periods, frequent interventional procedures, immobilization, the need to maintain specific positions, and the additional burden of donor site care were reported to further increase the complexity of care.

**Figure 3:**
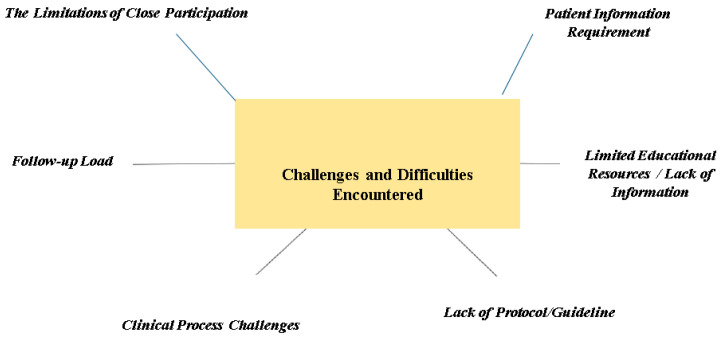
Display of theme 2. It was created by the authors during the analysis phase.

Participants also noted that the lack of standardized protocols and structured follow-up forms at the institutional level, variability in evaluation criteria among clinicians, and the absence of clearly defined numerical threshold values contribute to uncertainty in clinical decision-making processes. Limited educational opportunities related to flap care and reliance on experiential learning were also identified as significant challenges. In addition, insufficient involvement of patients and their relatives in the care process, along with the fact that responsibility for care largely rests with nurses, were cited as additional complicating factors. In this respect, Theme 2 highlights that flap care involves challenges not only from a clinical perspective but also from organizational and educational perspectives. The subthemes identified in this theme are shown in Figure 3.

#### 3.2.1. Follow-Up Load

This sub-theme includes codes related to the challenges of hourly monitoring, service intensity, bed occupancy, the need for continuous observation, and monitoring fatigue. Participants stated that the requirement for uninterrupted and frequent monitoring during the early period of flap care significantly increases both the workload and cognitive burden. It was emphasized that hourly assessments, especially within the first 24–48 h, make sustainability difficult under conditions of high service intensity. The increased frequency of monitoring makes it more challenging for nurses to simultaneously monitor multiple high-dependency patients and increases the risk of distraction. Participants noted that flap patients are considered a “patient group requiring high attention,” and that caring for these patients requires dedicated attention and time planning beyond routine ward tasks. It was further stated that this situation leads to mental fatigue and creates a continuous need to remain alert throughout the shift. Participant opinions indicate that the monitoring burden represents not only a physical workload but also a responsibility-driven cognitive load.

*“Hourly monitoring is really exhausting; it becomes difficult when there are other patients as well.”*

(N2)

*“When the ward is full, monitoring this frequently isn’t so easy; it adds extra workload.”*

(N4)

*“You constantly have the flap at the back of your mind, always wondering if something happened.”*

(N1)

*“The burden of monitoring is heavy, and it feels even harder, especially during the night shift.”*

(N6)

#### 3.2.2. Clinical Process Challenges

This subtheme encompasses prolonged fasting periods, frequent blood draws, difficulty with intravenous access, burden of donor site monitoring, stress related to revision surgery, challenges in caring for immobilized patients, risks associated with unconscious or inadequately informed patients, and difficulty in maintaining appropriate positioning. Participants stated that, in the care of flap patients, not only the flap site but also the overall clinical condition of the patient is complex, making care practices more challenging. It was noted that prolonged postoperative restrictions on oral intake, frequent laboratory monitoring, and difficulties with intravenous access further complicate the care process. Additionally, the need for immobilization in most flap patients, the requirement to maintain specific positioning, and the occurrence of unintentional patient movements were identified as factors that increase care-related risks. Participants stated that maintaining the patient’s position is critical for flap viability; however, this becomes challenging in the presence of discomfort, pain, and agitation. The requirement for simultaneous monitoring and care of the donor site was also highlighted as a factor that effectively doubles the care workload. The possibility of revision surgery was described as an additional source of stress. The need for urgent surgery intervention in cases of potential circulatory compromise renders the care process both high-risk and time-sensitive. Participants indicated that this situation creates pressure on both the patient and the healthcare team. Overall, this subtheme demonstrates that flap care is experienced not only as a technical monitoring process but also as a multidimensional clinical process involving position management, hemodynamic monitoring, interventional procedures, and the risk of complications.

*“It’s not just the flap; there’s also the donor area; we’re monitoring both regions at once.”*

(N5)

*“We constantly check the patient’s position because if they move, the flap might be affected.”*

(N3)

*“Being fasting for a long time is also difficult for the patient, and this impacts the care.”*

(N2)

*“When there’s a possibility of a revision surgery, everyone gets anxious.”*

(N7)

*“Sometimes it’s very difficult to find a vein.”*

(N4)

#### 3.2.3. Lack of Protocol/Guideline

This sub-theme includes the codes related to the lack of standardized protocols, insufficient institutional documentation in the form of non-standardized records, the absence of a common clinical language resulting in differences in assessment, and the lack of numerical criteria reflected in undefined risk thresholds. Participants emphasized that flap care practices are largely guided by individual experience, clinical culture, and physician input, and they highlighted the absence of a structured, step-by-step monitoring guide or checklist specific to nursing care. It was stated that this situation creates uncertainty in decision-making, especially among novice nurses, and leads to variability in assessment and timing of interventions, even among those working within the same clinic. The absence of clearly defined numerical threshold values, such as those related to temperature, color change, and capillary refill time, results in practices based on individual interpretation regarding when a situation is considered high risk and when the physician should be notified.

*“We don’t have a chart that shows the steps for the flap, we have general care forms, but they are not specific to the flap.”*

(N6)

*“Assessment varies from person to person. Those with experience know, but for beginners it’s hard to follow, there should be an indicator chart.”*

(N4)

*“Which color is normal, which one is risky? If there were a standard for these, everyone would speak the same language.”*

(N5)

#### 3.2.4. Limited Educational Resources/Lack of Information

This sub-theme includes the codes related to the lack of specific flap care training and insufficient in-house education. Most participants stated that they had not received structured training in flap care and had primarily learned through physicians, senior nurses, and clinical experience. The lack of formal training was described as a source of anxiety and difficulty, particularly during the early stages of care. It was noted that, due to limited or irregular institutional training opportunities, learning often progressed through individual effort and experiential practice.

*“I haven’t received any special flap training; I learned as I observed.”*

(N7)

*“There’s no specific training within the institution; we move forward with what the instructor explains.”*

(N5)

*“I have a wound care certificate, but it’s not specifically for flaps; I developed my skills through my own research.”*

(N6)

#### 3.2.5. Patient Information Requirement

This sub-theme encompasses the codes related to uncertainty regarding the care process, the need for education, and the provision of motivational support. Participants stated that the postoperative flap stage process is mostly unknown to patients and their relatives, which leads to fear, panic, and unrealistic expectations. Nurses play not only a clinical role during this period but also an intensive role in providing information and psychosocial support. It was emphasized that effective patient education improves the quality of care and facilitates patient compliance.

*“The patient and their relatives are unaware of the process, they may panic. Providing information is very important.”*

(N5)

*“Since it is a process they know nothing about, it’s necessary to put them at ease first.”*

(N5)

*“When families don’t know what to expect, their anxiety increases.”*

(N7)

#### 3.2.6. The Limitations of Close Participation

This subtheme consists of codes related to the limited involvement of families in the care process and feelings of discomfort due to changes in the patient’s appearance. Participants noted that changes in the patient’s appearance, especially in the head and neck region and in cases involving large tissue flaps, may negatively affect family engagement. Additionally, it was stated that families are often unable to participate in the care process due to the need for close monitoring, immobilization, and clinical restrictions. As a result, both the physical and emotional burden of care increase for nurses.

*“Families often cannot be involved in the process; all the care is left to us.”*

(N7)

*“Because the appearance changes, families can have difficulty approaching.”*

(N7)

*“Due to intensive monitoring, we cannot involve the patient’s relatives very much.”*

(N3)

### 3.3. Theme 3: Emotional and Professional Reflections

**Figure 4:**
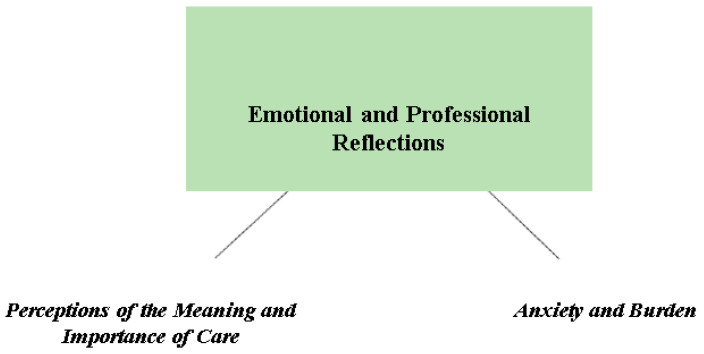
Display of theme 3. It was created by the authors during the analysis phase.

This theme demonstrates that flap care is not merely a technical and clinical follow-up process; it is also a multidimensional experience that creates significant emotional, cognitive, and professional impacts on nurses. Participants indicated that maintaining flap viability fosters a sense of professional satisfaction and accomplishment; in contrast, the risk of complications, sudden circulatory changes, and the need for revision procedures lead to considerable anxiety and stress. During the early postoperative period, uncertainty and the need for rapid intervention increase nurses’ state of constant vigilance and cognitive burden. Furthermore, changes in the patient’s appearance, the lengthy treatment process, and associated psychological effects create an additional empathetic burden for nurses, which in some cases renders the process emotionally exhausting. These findings reveal that flap care requires not only technical competence but also emotional resilience. The subthemes identified in this theme are shown in Figure 4.

#### 3.3.1. Perceptions of the Meaning and Importance of Care

This subtheme includes the codes related to satisfaction associated with a viable flap, the sense of professional fulfillment derived from patient recovery, and the perception of responsibility. Participants stated that findings indicating that the flap was healthy created professional satisfaction, and that being able to observe the outcomes of care in a tangible way was motivating. Observing signs of sustained circulation during the follow-up process provided nurses with a sense of relief and professional fulfillment, while maintaining flap viability was described as the most significant achievement of the care process. Some participants described flap monitoring as “seeing the reward of one’s labor” and “care with outcomes that can be directly observed.”

*“Flap’s being healthy provides spiritual satisfaction. When I hear the Doppler sound, I truly feel relieved; that sound means a lot to us.”*

(N1)

*“It is a care process with high responsibility but a beautiful outcome.”*

(N2)

*“If the flap we are monitoring progresses without problems, it makes us happy.”*

(N5)

#### 3.3.2. Anxiety and Burden

This sub-theme encompasses the stress associated with flap loss, demoralization in cases requiring revision, the impact of the patient’s appearance, exhaustion due to the prolonged nature of the process, and empathic burden. Participants emphasized that the flap monitoring process requires constant vigilance, and that even minor changes in circulation generate significant anxiety, particularly during the first 24–48 h, which were described as highly stressful. It was stated that signs such as a weakened or absent Doppler pulse, as well as changes in color and temperature that may require urgent intervention, keep nurses in a constant state of alert. When revision surgery is required, the morale of both the patient and the care team was reported to be negatively affected. Participants also noted that, because the flap is often located in visible areas, changes in the patient’s body image and associated psychological effects create an empathic burden for nurses. A lengthy hospitalization period, repeated surgeries, and care extending over several weeks were described as factors that increase the exhausting nature of the process from a professional perspective. Some participants indicated that the adaptation difficulties experienced by the patient can emotionally affect the caregiver as well, potentially increasing the risk of burnout.

*“When I can’t hear my flap, I get anxious and feel the need to check it repeatedly.”*

(N5)

*“It’s a long and exhausting process; sometimes we follow the same patient under these stressful conditions for weeks.”*

(N7)

*“When a patient needs a revision, our morale really drops down; knowing that the flap we worked hard for is at risk affects us as well.”*

(N3)

*“If the flap is on the patient’s face, their appearance changes a lot, and inevitably, we feel the emotion the patient is experiencing too.”*

(N2)

*“In the first days, my mind is constantly on the patient, and I frequently go and check to see if anything has changed.”*

(N9)

### 3.4. Theme 4: Suggestions for Improving Care

**Figure 5:**
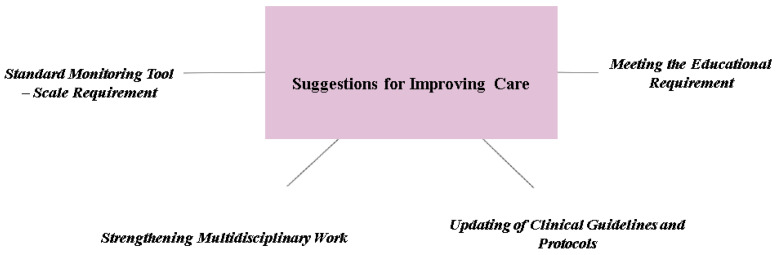
Display of theme 4. It was created by the authors during the analysis phase.

The participants’ views not only described current care practices but also offered concrete and systematic suggestions for improving the flap care process. These suggestions focused on the need for a standardized monitoring tool, strengthened multidisciplinary collaboration, and the identification of deficiencies in clinical guidelines and training. Participants indicated that flap monitoring was largely conducted based on individual interpretation, resulting in variability in clinical decision-making processes. Therefore, it was emphasized that a structured monitoring form containing numerical threshold values and clear intervention criteria was necessary. It was also stated that communication within the team should be strengthened and that collaboration between surgeons and nurses should be more systematically organized. Additionally, the limited availability of training opportunities and up-to-date clinical guidelines specific to flap care remains an important area requiring improvement. These findings highlight the need to shift from practices based on individual experience to standard and evidence-based approaches in flap care. The subthemes identified in this theme are shown in Figure 5.

#### 3.4.1. Standard Monitoring Tool—Scale Requirement

This sub-theme includes the need for a color scale, temperature threshold values, Doppler scoring, capillary refill grading, bleeding/hematoma recording, pain assessment, a risk scoring system, an algorithm defining when to notify the physician, positioning guidelines, donor site monitoring components, nutritional status, and pressure ulcer risk assessment. Participants stated that flap monitoring is largely observational and open to interpretation, which complicates both intra-team communication and clinical decision-making processes. Therefore, the need for a follow-up form or scoring system based on numerical thresholds and grading is frequently emphasized. It was noted that the presence of visual and numerical indicators to facilitate the distinction between normal and high-risk findings, particularly for novice nurses, would enhance patient safety. Participants also expressed that not only the flap site but also the donor site, nutritional status, and pressure ulcer risk should be included in the same monitoring tool. Participant feedback indicates that the proposed tool is expected to function not only as a recording system but also as a clinical decision support system.

*“There is a form, but it is not standard; one writes purple, another writes pink. It would be better if there was a numerical equivalent.”*

(N3)

*“At what temperature is it risky, at what temperature is it normal? There should be such an indicator.”*

(N4)

*“I need to know under what conditions I should call the physician? It should be like an algorithm.”*

(N3)

*“Color, temperature, Doppler, capillary refill… There could be a scoring system for all of them.”*

(N5)

*“The donor site should definitely be included in the follow-up form as well.”*

(N8)

#### 3.4.2. Strengthening Multidisciplinary Work

This sub-theme encompasses the importance of teamwork and the need for collaborative follow-up, including codes related to surgeon–nurse collaboration. Participants stated that flap care is multidisciplinary by its nature; however, in practice, some processes are physician-centered, and nurses can be left out of the process at certain stages. It was noted that, particularly during dressing and assessment, greater involvement of nurses would be beneficial in terms of infection control and continuity of care. It was emphasized that establishing a common assessment language within the team and implementing regular feedback mechanisms would improve the quality of care. These findings indicate that, while the participants support the multidisciplinary structure, they also expect the nursing role to be strengthened.

*“The team is very important; everyone needs to stay alert.”*

(N3)

*“The surgeon and nurse should monitor together; there should be a shared system.”*

(N5)

*“When the dressing is done entirely by the assistant, we are left out of the process.”*

(N8)

#### 3.4.3. Updating of Clinical Guidelines and Protocols

In this sub-theme, participants stated that current clinical guidelines do not provide detailed, flap-specific instructions and that most practices are carried out based on experience. The need for standardized care guidelines at the national or institutional level to support the consistency of practice and training processes was emphasized. Participants considered such guidelines particularly important for defining risk indicators and clarifying the timing of interventions.

*“Something like a care manual would be very instructive for the nurse.”*

(N3)

*“If there were guidelines, everyone would do the same thing.”*

(N5)

#### 3.4.4. Meeting the Educational Requirement

In this sub-theme, participants stated that educational opportunities specific to flap care are limited and that most learning occurs through clinical experience. It was emphasized that regular in-house training programs, hands-on workshops, and case-based teaching would be beneficial. It was suggested that novice nurses should go through a period of observation and supervised practice before assuming direct responsibility. These findings indicate that participants perceive a need for institutionalized educational models in flap care.

*“Beginners should watch first; training should be increased.”*

(N5)

*“There is no special flap training; we learn most things by observing.”*

(N2)

*“I learned many things through my own research.”*

(N6)

## 4. Discussion

The qualitative findings obtained within the scope of this study provide an in-depth understanding of nursing approaches and clinical decision-making mechanisms related to the critical care process following free tissue flap transplantation, as reflected in nurses’ experiences. In the literature, early clinical monitoring after free flap surgery is considered the “gold standard” and is defined as the serial evaluation of the flap’s vascular status using visual and tactile parameters such as color, capillary refill, temperature, and tissue turgor [2,6,17,18]. In this discussion section, nurses’ clinical experiences are evaluated from an analytical perspective considering current protocols and technological developments in the literature.

### 4.1. Clinical Monitoring and Evaluation in the Care Process

According to the findings of the study, nurses’ identification of the first 24–48 h as a critical period and their practice of hourly monitoring are fully consistent with fundamental management principles described in the literature. Previous studies emphasize that the majority of vascular complications occur within the first 72 h, with the first 24 h representing the most critical period for flap survival [3,6,19]. It has been reported that hourly monitoring is standard practice, especially during the first 24–48 h, and that this frequency is important for the early detection of flap compromise [2,10,20]. The emphasis on immobilization and close monitoring observed in the findings of this study is consistent with the literature highlighting the importance of protecting flap tissue from thrombosis, twisting, or external compression [2,3]. Furthermore, close monitoring of vital signs is an integral part of the holistic care of the microsurgery patient, considering the direct impact of systemic perfusion on flap circulation [2,19,20]. Rapid notification of suspicious findings and effective communication with the surgeon are critical for successful flap salvage. The literature indicates that when a vascular crisis is identified early, intervention within the first 24 h can maintain survival rates at 70–80%, whereas delays may reduce this rate to below 30% [2,10]. The emphasis on early notification in the present findings is consistent with evidence showing that successful flap outcomes are associated with earlier detection compared to failed cases [10]. In this process, the role of nurses is vital for the early detection of complications and timely notification of the surgical team [2,9,10,21].

In the study, nurses’ focus on parameters such as color, temperature, capillary refill time, and edema/hematoma control is consistent with non-invasive clinical parameters widely accepted in the literature [17,20,22]. The literature indicates that, in cases of arterial insufficiency, the flap becomes pale and capillary refill time is prolonged; in contrast, in venous congestion, the flap assumes a purple-blue coloration, tissue turgor increases, and capillary refill time is shortened, typically to less than two seconds [23,24]. The critical importance of hematoma control is also well supported in the literature, which defines hematoma as one of the most significant factors causing flap loss due to compression of the vascular pedicle. The use of Doppler assessment as an auxiliary tool is also a common practice; however, the literature warns that the presence of Doppler signals does not always indicate adequate perfusion [6,17]. In this study, as noted by the nurses, methods such as Doppler monitoring, non-contact thermometer, and photographic monitoring are widely discussed in the literature. The handheld Doppler is one of the most commonly used auxiliary methods [6,10,20,25]. Non-contact temperature measurement is also consistent with the literature, which considers differences in temperature between the flap and surrounding tissue as an indicator of potential complications [17,24].

The finding that nurses’ decision-making processes are largely based on experience is consistent with the concept of a “trained eye” emphasized in the literature [5,9]. Clinical assessment is inherently subjective and may show differences between observers [2,6,26]. As also observed in the present study, this situation leads experienced nurses to rely on clinical intuition.

### 4.2. Challenges and Difficulties Encountered

The findings of this study regarding the need for hourly flap monitoring and continuous observation are consistent with the “intensive labor” care process described in the literature [27]. According to the experiences of the participating nurses, ward intensity and workload indicate that the care process is highly demanding. The finding that nurses have limited access to training programs specific to flap care and that learning is based on experience is consistent with reports of “nursing knowledge deficiency” in the literature [9,10]. Studies have shown that nurses’ initial knowledge levels can be insufficient, especially in recognizing signs of venous congestion, but structured training can lead to rapid improvement [9,10]. Furthermore, the findings related to process uncertainty and patient and family anxiety align with the understanding that patients undergoing head and neck and microsurgical procedures experience a psychosocially exhausting process [20]. The literature also indicates that preoperative patient education is often inadequate and that patients wish to be informed about both favorable and adverse scenarios, including the possibility of revision surgery [28].

The lack of standard follow-up algorithms identified from the experiences of the nurses participating in this study and the differences in practices between institutions are directly related to the problem referred to in the literature as the “standardization gap” [10]. The literature confirms that flap monitoring techniques are largely surgeon-dependent and show significant differences between institutions [2,9,20]. The absence of quantitative threshold values and the subjective nature of clinical evaluation make the decision-making process particularly challenging for less experienced personnel [9].

### 4.3. Emotional and Professional Reflections

The finding of this study that a healthy flap generates a sense of satisfaction among nurses and that maintaining flap viability is perceived as an indicator of care success is consistent with evidence in the literature showing that follow-up protocols conducted by trained nurses are associated with high success rates and positively influence nurses’ professional self-confidence and self-esteem [21]. In this context, flap survival can be considered not only a clinical outcome but also tangible evidence of the quality of nursing care. The stress and constant vigilance experienced by nurses in cases of Doppler signal loss are directly related to the “high-risk monitoring burden” described in the literature. The literature indicates that the subjective nature of clinical assessment, including parameters such as color, turgor, and temperature, places considerable pressure, particularly on less experienced staff [9,21]. The stress associated with the possibility of revision surgery, as identified in this study, reflects concerns regarding the accuracy of decisions made during a vascular crisis. Exhaustion resulting from continuous monitoring may be considered a natural consequence of the labor-intensive nature of microsurgical care.

The theme of being affected by changes in appearance and establishing an empathetic connection, as identified in this study, is consistent with issues reported in the literature, including changes in body image and social interactions, disfigurement, and the psychological distress experienced by patients who have undergone flap surgery [28]. Nurses, who closely observe patients’ changes in body image and experiences, carry a significant empathetic burden. The connection that healthcare professionals establish with the patient’s emotional journey turns microsurgery care into not just a technical monitoring process, but also an intense process of emotional labor.

### 4.4. Suggestions for Improving Care

The literature recommends the use of standardized protocols and objective monitoring tools such as tissue oximetry and implantable Doppler devices to reduce subjectivity. Variability in protocols across institutions and the lack of standardization contribute to a persistent standardization gap [10].

The need for a standardized monitoring tool, including components such as a color scale, temperature threshold values, and capillary refill grading, as emphasized in the findings of this study, is directly related to the lack of objective data reported in the literature. Although 1–10 scoring scales have been proposed for the assessment of flap color, their use has not become widespread [6]. However, the demand for numerical threshold values and a risk scoring system identified in this study reflects the need for more concrete tools to reduce reliance on individual interpretation in clinical decision-making. The challenges associated with interpreting Doppler signals and the risk of false-positive findings, as reported in the literature [6,17], further support the recommendation for standardized recording systems and structured assessment tools.

The lack of specialized education in flap care and the reliance on experience-based learning through master-apprentice relationships are also identified in the literature as significant problem areas [9,10]. Studies indicate that nurses may initially show low success in recognizing the signs of venous congestion; however, structured training leads to substantial improvement [9,10]. In this study, the proposed structured training programs and certification processes are consistent with recommendations in the literature aimed at standardizing nursing competencies and ensuring safe care.

### 4.5. Limitations and Strengths

This study has several limitations. First, the data were collected based on participants’ self-reports, which may have introduced bias depending on individual experiences and perceptions. Although the varying levels of clinical experience among participants increased the diversity of the findings, it may also have contributed to differences in interpretation. Furthermore, this study is limited to the views of nurses and does not include the perspectives of other members of the multidisciplinary team such as physicians and the anesthesia staff.

Nevertheless, this study has significant strengths. It contributes to the limited body of qualitative research by providing an in-depth exploration of the free tissue flap care process through nurses’ experiences. The use of semi-structured interviews enabled participants to express their experiences in a detailed and comprehensive manner. In addition, a systematic approach was followed in the thematic analysis, and a high level of inter-researcher agreement was achieved, supporting the reliability of the findings. By directly highlighting the challenges and needs encountered in clinical practice, this study offers important insights for the development of standardized monitoring tools, training programs, and clinical guidelines for flap care.

## 5. Conclusions

This study comprehensively examined the clinical, emotional, and organizational aspects of flap care by qualitatively exploring the experiences of nurses involved in free tissue flap care. The findings indicate that nurses conduct an intensive, multidimensional, and highly attentive monitoring process that involves substantial responsibility and cognitive load.

It was determined that flap monitoring is largely based on experience and subjective assessments, and that the lack of standardized protocols and structured monitoring tools creates a significant gap in the care process. Nevertheless, nurses play a critical role in the early detection of complications through rapid recognition, early notification, and effective team communication. However, the constant vigilance required, the risk of complications, and the prolonged care process impose a considerable emotional burden and may contribute to professional burnout among nurses.

In line with the research findings, enhancing the quality of care in flap management requires the development of standardized and structured monitoring tools based on numerical thresholds, the strengthening of multidisciplinary collaboration, the updating of clinical guidelines, and the establishment of systematic training programs for nurses. In addition, promoting informational approaches that support the active participation of patients and their relatives in the care process may improve care effectiveness. In conclusion, nurses are at the center of the free flap care process, and the standardization and support of care are critical for increasing patient safety and the quality of care. This study contributes to increasing the visibility of nursing practices in flap care and provides a scientific basis for the development of training programs, clinical guidelines, and monitoring models in this field.

## Figures and Tables

**Table 1:** Descriptive data of the participants.

Participant No.	Age	Gender	Professional Experience/Years	Whether They Received Special Training Related to Flap Care
1	26	Female	2	By the department instructor
2	26	Female	3	By the department instructor
3	29	Female	7	By the department instructor
4	30	Female	6	By the department instructor
5	28	Female	5	By the department instructor
6	38	Female	14	No
7	43	Female	20	No
8	25	Male	6	No
9	31	Female	8	By the department instructor

**Table 2:** Overview of themes and subthemes.

Main Themes	Subthemes
Theme 1. Clinical Monitoring and Evaluation in the Care Process	Close monitoring during critical hours; Evaluation based on clinical indicators; Experience-based decision making: knowledge and experience used in the observation process; Clinical decision-making and intervention process; Monitoring tools and techniques used
Theme 2. Challenges and Difficulties Encountered	Follow-up load; Clinical process challenges; Lack of protocol/guideline; Limited educational resources/lack of information; Patient information requirement; The limitations of close participation
Theme 3. Emotional and Professional Reflections	Perceptions of the meaning and importance of care; Anxiety and burden
Theme 4. Suggestions for Improving Care	Standard monitoring tool/scale requirement; Strengthening multidisciplinary work; Updating of clinical guidelines and protocols; Meeting the educational requirement

Note. The themes and subthemes were generated by the authors during the qualitative data analysis process.

## Data Availability

The data presented in this study are available on request from the corresponding author due to privacy.

## Abbreviation

The following abbreviation is used in this manuscript:

COREQ	The Consolidated Criteria for Reporting Qualitative Research
